# Vesicular Stomatitis Virus-Based Vaccines Protect Nonhuman Primates against *Bundibugyo ebolavirus*

**DOI:** 10.1371/journal.pntd.0002600

**Published:** 2013-12-19

**Authors:** Chad E. Mire, Joan B. Geisbert, Andrea Marzi, Krystle N. Agans, Heinz Feldmann, Thomas W. Geisbert

**Affiliations:** 1 Galveston National Laboratory, University of Texas Medical Branch, Galveston, Texas, United States of America; 2 Department of Microbiology and Immunology, University of Texas Medical Branch, Galveston, Texas, United States of America; 3 Laboratory of Virology, Division of Intramural Research, National Institute of Allergy and Infectious Diseases, National Institutes of Health, Hamilton, Montana, United States of America; Tulane School of Public Health and Tropical Medicine, United States of America

## Abstract

Ebola virus (EBOV) causes severe and often fatal hemorrhagic fever in humans and nonhuman primates (NHPs). Currently, there are no licensed vaccines or therapeutics for human use. Recombinant vesicular stomatitis virus (rVSV)-based vaccine vectors, which encode an EBOV glycoprotein in place of the VSV glycoprotein, have shown 100% efficacy against homologous *Sudan ebolavirus* (SEBOV) or *Zaire ebolavirus* (ZEBOV) challenge in NHPs. In addition, a single injection of a blend of three rVSV vectors completely protected NHPs against challenge with SEBOV, ZEBOV, the former *Côte d'Ivoire ebolavirus*, and Marburg virus. However, recent studies suggest that complete protection against the newly discovered *Bundibugyo ebolavirus* (BEBOV) using several different heterologous filovirus vaccines is more difficult and presents a new challenge. As BEBOV caused nearly 50% mortality in a recent outbreak any filovirus vaccine advanced for human use must be able to protect against this new species. Here, we evaluated several different strategies against BEBOV using rVSV-based vaccines. Groups of cynomolgus macaques were vaccinated with a single injection of a homologous BEBOV vaccine, a single injection of a blended heterologous vaccine (SEBOV/ZEBOV), or a prime-boost using heterologous SEBOV and ZEBOV vectors. Animals were challenged with BEBOV 29–36 days after initial vaccination. Macaques vaccinated with the homologous BEBOV vaccine or the prime-boost showed no overt signs of illness and survived challenge. In contrast, animals vaccinated with the heterologous blended vaccine and unvaccinated control animals developed severe clinical symptoms consistent with BEBOV infection with 2 of 3 animals in each group succumbing. These data show that complete protection against BEBOV will likely require incorporation of BEBOV glycoprotein into the vaccine or employment of a prime-boost regimen. Fortunately, our results demonstrate that heterologous rVSV-based filovirus vaccine vectors employed in the prime-boost approach can provide protection against BEBOV using an abbreviated regimen, which may have utility in outbreak settings.

## Author Summary

Ebola viruses (EBOV), of which there are five species, are categorized as Category A Priority Pathogens and Tier 1 Select Agents by several US Government agencies as a result of their high mortality rates and potential for use as agents of bioterrorism. Currently, there are no vaccines or therapeutics approved for human use. Replication-competent, recombinant vesicular stomatitis virus (rVSV) vectors expressing filovirus glycoproteins (GP), in place of the VSV glycoprotein have shown promise in lethal nonhuman primate (NHP) models of filovirus infection as both single injection preventive vaccines and as post-exposure treatments. The recent outbreak of the fifth recognized EBOV species, *Bundibugyo ebolavirus* (BEBOV), demonstrates the need for vaccines that can be rapidly deployed to combat an outbreak of a new filovirus species. To date, rVSV-filovirus GP-based vaccines have only been able to protect against challenge with a homologous species of EBOV. Here, we show that the two heterologous rVSV-based filovirus vaccines available at the time of the original BEBOV outbreak can protect NHPs against BEBOV challenge using a short prime-boost vaccination strategy. While the prime-boost strategy was successful, a single injection blended vaccination strategy with the same vaccine vectors failed to provide protection. These data suggest that an abbreviated prime-boost regimen of 36 days may have utility for quickly responding to outbreaks caused by new species of EBOV.

## Introduction

The viruses in the family *Filoviridae* and within the genera *Ebolavirus* (EBOV) and *Marburgvirus* (MARV) cause severe and often fatal hemorrhagic fever (HF) in humans and nonhuman primates (NHPs) [1], [2]. Case fatality rates with these viruses range from 23–90% depending on the strain and/or species. The EBOV genus is diverse and, as of 2007, consisted of four species: *Sudan ebolavirus* (SEBOV), *Zaire ebolavirus* (ZEBOV), *Côte d'Ivoire ebolavirus* (CIEBOV), and *Reston ebolavirus* (REBOV). A fifth species, *Bundibugyo ebolavirus* (BEBOV) was discovered during an outbreak in Uganda during 2007/08 [3]. Before 2012, the EBOV genus had accounted for at least 22 outbreaks dating back to 1976 with 18 of these occurring within the last 20 years [4]. In 2012 there were two separate outbreaks of EBOV; SEBOV in Uganda [5] and BEBOV in the Democratic Republic of Congo (DRC) [6]. The increased frequency of EBOV outbreaks together with the potential for deliberate misuse has increased public health concerns regarding filoviruses. Case fatality rates frequently range between 70% and 90% in ZEBOV outbreaks, 50–55% for SEBOV episodes, and 40–48% for BEBOV outbreaks. CIEBOV caused deaths in chimpanzees and a severe nonlethal human infection in a single case in the Republic of Côte d'Ivoire in 1994 [7]. REBOV is highly lethal for macaques but is not thought to cause disease in humans [8].

Presently, there are no licensed vaccines or post-exposure treatments available for human use; however, there are at least seven different vaccine candidates that have shown the potential to protect NHPs from lethal EBOV and/or MARV infection using platforms based on DNA vectors, recombinant Adenovirus (rAd) vectors, combined DNA/rAd vectors, virus-like particles (VLPs), alphavirus replicons, recombinant human parainfluenza virus 3 (rHPIV3), and recombinant vesicular stomatitis virus (rVSV) [9], [10], [11], [12], [13], [14], [15], [16], [17], [18], [19], [20], [21], [22], [23], [24], [25], [26], [27], [28], [29]. The EBOV vaccine systems rely on antigens specific for each species of virus to provide protection against lethal challenge in NHP models; however, there is no description of a vaccine approach yet that can provide 100% single immunization cross species protection against challenge with an emerging filovirus such as BEBOV.

The rVSV filovirus vaccine platform, reported on herein, relies on the filovirus glycoprotein (GP) as the immunizing antigen [12]. Current data suggest that the GP from each filovirus species can only protect against homologous challenge when using the rVSV vaccine platform as a single injection [16], [25]. Cross-protection with the rVSV vaccines has been achieved using a blended vaccination strategy where a mixture of three separate vaccine vectors, rVSV-MARV-GP, rVSV-ZEBOV-GP, and rVSV-SEBOV-GP were able to protect against separate challenge with either MARV, ZEBOV, CIEBOV, or SEBOV in NHPs [16]. Although cross-protection was achieved using this blended vaccination strategy against challenge of known species of EBOVs, the BEBOV outbreak in 2007 offered a new challenge to develop a strategy to protect against an emerging species of EBOV using existing vaccines that were available at the time of the outbreak. This strategy was tested in cynomolgus macaques using two different vaccine platforms against heterologous challenge with BEBOV; the DNA/rAd platform [23] and the rVSV-filovirus-GP platform [25] where the mortality rate for BEBOV in cynomolgus macaques was found to be 66 to 75%. The study using the DNA/rAd platform consisted of four ZEBOV-GP/SEBOV-GP DNA vaccinations given over the course of 14 weeks and a boost vaccination consisting of the ZEBOV rAd5 GP ZEBOV vector 12 months after the final DNA vaccination. This strategy, although long and complicated, was able to confer 100% protection to the NHPs used in the study [23]. In contrast, the rVSV vaccine strategy employed to protect against heterologous challenge with BEBOV was a single vector strategy. The NHPs in this study were vaccinated with rVSV-ZEBOV-GP or rVSV-CIEBOV-GP separately and challenged with BEBOV 28 days after vaccination. While the rVSV-CIEBOV-GP vector did not provide any additional protection when compared to mock-vaccinated control NHPs in the study (33% survival), the rVSV-ZEBOV-GP vaccine conferred 75% survival [25]. This result was surprising when one considers CIEBOV is more genetically related to BEBOV when compared to ZEBOV [3]. The use of SEBOV and ZEBOV GP as antigens to confer 100% protection against cross species challenge with BEBOV using the DNA/rAd strategy [23] suggested that if the rVSV-SEBOV-GP vaccine was used in combination with rVSV-ZEBOV-GP the cross species protection using the rVSV system would increase from 75% with just rVSV-ZEBOV-GP [25] to 100% protection.

Here, we evaluated the utility of combining rVSV-SEBOV-GP and rVSV-ZEBOV-GP vectors using either a single injection blended vaccination approach or in a prime-boost regimen against heterologous BEBOV challenge in cynomolgus macaques. Furthermore, we assessed the ability of a single injection of a newly developed homologous rVSV-BEBOV-GP vaccine vector to provide protection against homologous BEBOV challenge.

## Materials and Methods

### Ethics statement

Healthy, adult cynomolgus macaques (*Macaca fascicularis*) were handled in Animal BSL-2 and BSL-4 containment space in the Galveston National Laboratory (GNL) at the University of Texas Medical Branch (UTMB), Galveston, Texas. Research was conducted in compliance with the Animal Welfare Act and other federal statutes and regulations relating to animals and experiments involving animals, and adhered to principles stated in the Eighth edition of the Guide for the Care and Use of Laboratory Animals, National Research Council, 2013. The facility where this research was conducted (UTMB) is fully accredited by the Association for the Assessment and Accreditation of Laboratory Animal Care International and has an approved OLAW Assurance #A3314-01. Research was conducted under animal protocol number 1011057 approved by the UTMB Institutional Animal Care and Use Committee (IACUC). All steps were taken to ameliorate the welfare and to avoid the suffering of the animals in accordance with the “Weatherall report for the use of nonhuman primates” recommendations. Animals were housed in adjoining individual primate cages allowing social interactions, under controlled conditions of humidity, temperature, and light (12-hour light/12-hour dark cycles). Food and water were available ad libitum. Animals were monitored (pre- and post-infection) and fed commercial monkey chow, treats and fruit twice daily by trained personnel. Environmental enrichment consisted of commercial toys. All procedures were conducted by trained personnel under the oversight of an attending veterinarian and all invasive clinical procedures were performed while animals were anesthetized. Endpoint criteria was specified and approved by the UTMB IACUC.

### rVSV vaccine vectors and challenge virus

**Figure 1:**
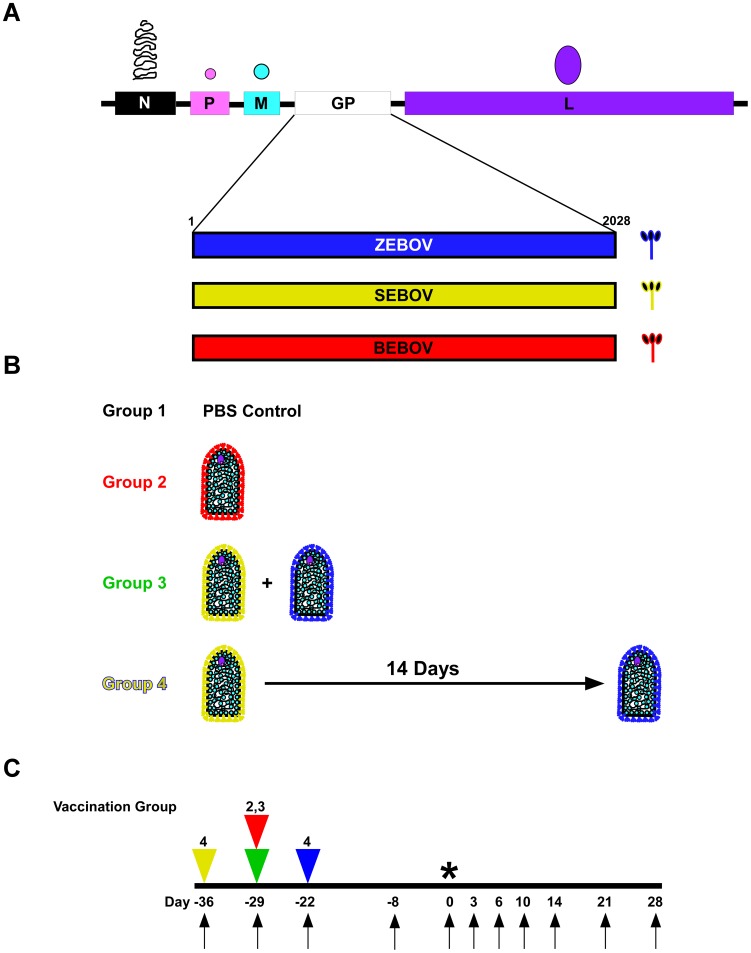
BEBOV cross-protection study design. (A) Diagram of rVSV genome for each vaccine used in this study. N; nucleoprotein, P; phosphoprotein, M; matrix protein, GP; filovirus glycoprotein (ZEBOV (blue), SEBOV (yellow), or BEBOV (red)), L; large polymerase protein. (B) Depiction of the vaccine groups: Group 1 (PBS only control, black), Group 2 (rVSV-BEBOV-GP only, red), Group 3 (rVSV-SEBOV-GP plus rVSV-ZEBOV-GP, green), and Group 4 (rVSV-SEBOV-GP only, then rVSV-ZEBOV-GP 14 days post, yellow/blue). (C) Flow chart showing the days of vaccination (triangles), days of sampling (arrows), and day of challenge (*). The yellow triangle represents the first vaccination phase of Group 4 where the animals were vaccinated with rVSV-SEBOV-GP, the red and green triangles represent the day Groups 2 (red) and 3 (green) were vaccinated, and the blue triangle represents the day of rVSV-ZEBOV-GP vaccination in Group 4.

The rVSV-filovirus GP-vectors, rVSV-ZEBOV-GP (strain Mayinga), rVSV-SEBOV-GP (strain Boniface), and rVSV-BEBOV-GP (Fig. 1A) were recovered from cDNA as previously described [30], [31]. BEBOV, strain 200706291, was isolated from a fatal human case in western Uganda during the outbreak in 2007 [3]. The challenge stock of BEBOV used in this study was propagated on Vero E6 cells twice making this a passage 2 virus. The viral genomes from this stock were Sanger-sequenced across the GP editing site and it was confirmed that the sequence from bases 6900 to 6907 was wild-type BEBOV (Accession: NC 014373.1). The BEBOV challenge stock was kindly provided by Dr. Thomas G. Ksiazek.

The rVSV-filovirus-GP vector preparations and BEBOV challenge virus stocks were assessed for the presence of endotoxin using The Endosafe®-Portable Test System (PTS) (Charles River, Wilmington, MA). Virus preparations were diluted 1∶10 in Limulus Amebocyte Lysate (LAL) Reagent Water (LRW) per manufacturer's directions and endotoxin levels were tested in LAL Endosafe®-PTS cartridges as directed by the manufacturer. Each preparation was found to be below detectable limits while positive controls showed that the tests were valid.

### Immunization and challenge

Twelve, healthy, filovirus-naïve, adult (5 to 12 kg), male cynomolgus macaques (*Macaca fascicularis*) were randomized into four different groups of three animals each (Groups 1, 2, 3, and 4; Figure 1B). Animals were vaccinated by intramuscular (i.m.) injection of an identical volume of PBS (Group 1), ∼2×10^7^ plaque-forming units (PFU) of rVSV-BEBOV-GP (Group 2), or ∼1×10^7^ PFU of rVSV-SEBOV-GP and ∼1×10^7^ PFU of rVSV-ZEBOV-GP (Groups 3 and 4). While Groups 3 and 4 received the same vaccine vectors the dosing regimens were different, as shown in Figure 1B and C. Group 3 received a single inoculation that was an equal blend of the two vaccines. Group 4 received two inoculations, the rVSV-SEBOV-GP vaccine first and the rVSV-ZEBOV-GP vaccine 14 days later. Four (Groups 1, 2, and 3) or 5 (Group 4) weeks after the initial vaccination, all animals were challenged i.m. with 1,000 PFU of BEBOV.

Animals were monitored for clinical signs of illness (temperature, weight loss, changes in blood count, and blood chemistries) during the vaccination and BEBOV challenge portions of the study. Viremia was analyzed after vaccination and challenge. Physical exams were given when blood was collected on days of vaccination and 8 days before challenge and on days 0, 3, 6, 10, 14, 21, and 28 post-challenge (Fig. 1C).

### Hematology and serum biochemistry analysis

Total white blood cell counts, white blood cell differentials, red blood cell counts, platelet counts, hematocrit values, total hemoglobin concentrations, mean cell volumes, mean corpuscular volumes, and mean corpuscular hemoglobin concentrations were analyzed from blood collected in tubes containing EDTA using a laser based hematologic analyzer (Beckman Coulter, Brea, CA). Serum samples were tested for concentrations of albumin, amylase, alanine aminotransferase (ALT) aspartate aminotransferase (AST), alkaline phosphatase (ALP), gamma-glutamyltransferase (GGT), glucose, cholesterol, total protein, total bilirubin (TBIL), blood urea nitrogen (BUN), creatine (CRE), and C-reactive protein (CRP) by using a Piccolo point-of-care analyzer and Biochemistry Panel Plus analyzer discs (Abaxis, Sunnyvale, CA).

### Detection of viremia

RNA was isolated from whole blood utilizing the Viral RNA mini-kit (Qiagen) using 100 µl of blood into 600 µl of buffer AVL. Primers/probe targeting the GP gene of BEBOV were used for quantitative real-time PCR (qRT-PCR) as used previously [25] with the probe used here being 6-carboxyfluorescein (6FAM)-5′ AGGCTTCCCTCGCTGCCGTTATG 3′-6 carboxytetramethylrhodamine (TAMRA) (Life Technologies). BEBOV RNA was detected using the CFX96 detection system (BioRad Laboratories, Hercules, CA) in One-step probe qRT-PCR kits (Qiagen) with the following cycle conditions: 50°C for 10 minutes (min), 95°C for 10 seconds (s), and 40 cycles of 95°C for 10 s and 59°C for 30 s. Threshold cycle (*CT*) values representing BEBOV genomes were analyzed with CFX Manager Software, and data are shown as + or − for genome equivalents (GEq) above or below 3.0 log_10_ respectively. To create the GEq standard, RNA from BEBOV stocks was extracted and the number of BEBOV genomes was calculated using Avogadro's number and the molecular weight of the BEBOV genome. Virus titration was performed by plaque assay with Vero E6 cells from all serum samples. Briefly, increasing 10-fold dilutions of the samples were adsorbed to Vero E6 monolayers in duplicate wells (200 µl); the limit of detection was 25 PFU/ml.

### Humoral immune response

Serum collected at indicated time points (Fig. 1C, vertical arrows) was tested for cross-reactive immunoglobulin G (IgG) antibodies against SEBOV, ZEBOV, and BEBOV. Enzyme-linked immunosorbent assay (ELISA) using purified virus-like particles (VLPs) containing VP40 and GP antigen for the appropriate filovirus, was used to detect cross-reactive IgG. VLPs were produced as previously described [32], with the exception of using baby hamster kidney (BHK) cells to produce the particles. Species specific VLPs were detergent lysed in 0.01% Triton-X 100-PBS and 1 µg of protein was used to coat the 96 well ELISA plates (Nunc). The serum samples were assayed at 4-fold dilutions starting at a 1∶100 dilution in ELISA diluents (1% heat inactivated fetal bovine serum (HI-FBS), 1×PBS, and 0.2% Tween-20). Samples were incubated for 1 hour at room temperature, removed, and plates were washed. Wells were then incubated at room temperature for 1 hour with anti-monkey IgG conjugated to horseradish peroxidase (Fitzgerald Industries International) at a 1∶2500 dilution. These wells were washed and then incubated with 2,2′-azine-di(3ethylbenzthiazoline-6-sulfonate) peroxidase substrate system (KPL) and read for dilution endpoints at 405 nm on a microplate reader (Molecular Devices Emax system). Statistics were calculated for ELISA IgG titers utilizing GraphPad Prism 5 software by using a 2way ANOVA analysis comparing treatments and times between all groups.

Neutralizing antibody titers were determined by performing plaque reduction neutralization titration assays (PRNT). Briefly, Vero cells were seeded into 6 well plates to generate a confluent monolayer on the day of infection. Serum dilutions were prepared in DMEM and 100 µL were incubated with ∼100 pfu of rVSV-BEBOV-GP in a total volume of 200 µL. Media was removed from cells, the serum–virus mixture was added and samples were incubated for 60 min at 37°C. The mixture was removed from the cells and 2 ml of 0.9% agaraose EMEM (5% FBS v/v) was overlayed on wells. Cells were observed 72 hours post-incubation and plaques were counted. The neutralizing antibody titer of a serum sample was considered positive at a dilution showing a ≥50% reduction (PRNT_50_) compared with the virus control without serum.

## Results

### The humoral immune response to BEBOV GP after immunization with rVSVs expressing heterologous glycoproteins

To evaluate whether a homologous monovalent vaccine could protect against BEBOV and whether or not we could achieve cross-protection against BEBOV with heterologous vaccines available at the time of the original BEBOV outbreak [3], we used the cynomolgus macaque BEBOV NHP model [23], [25]. In this study, we used four separate vaccination groups of NHPs as shown in Figure 1B. Group 1 (PBS) was a negative control group, Group 2 (BEBOV) was an internal control group vaccinated with a rVSV-BEBOV-GP vaccine, Group 3 (Blend) was vaccinated with an equal blend of rVSV-SEBOV-GP and rVSV-ZEBOV-GP in a single inoculation, and Group 4 (Boost) was vaccinated with rVSV-SEBOV-GP first and 14 days later vaccinated with rVSV-ZEBOV-GP.

**Figure 2:**
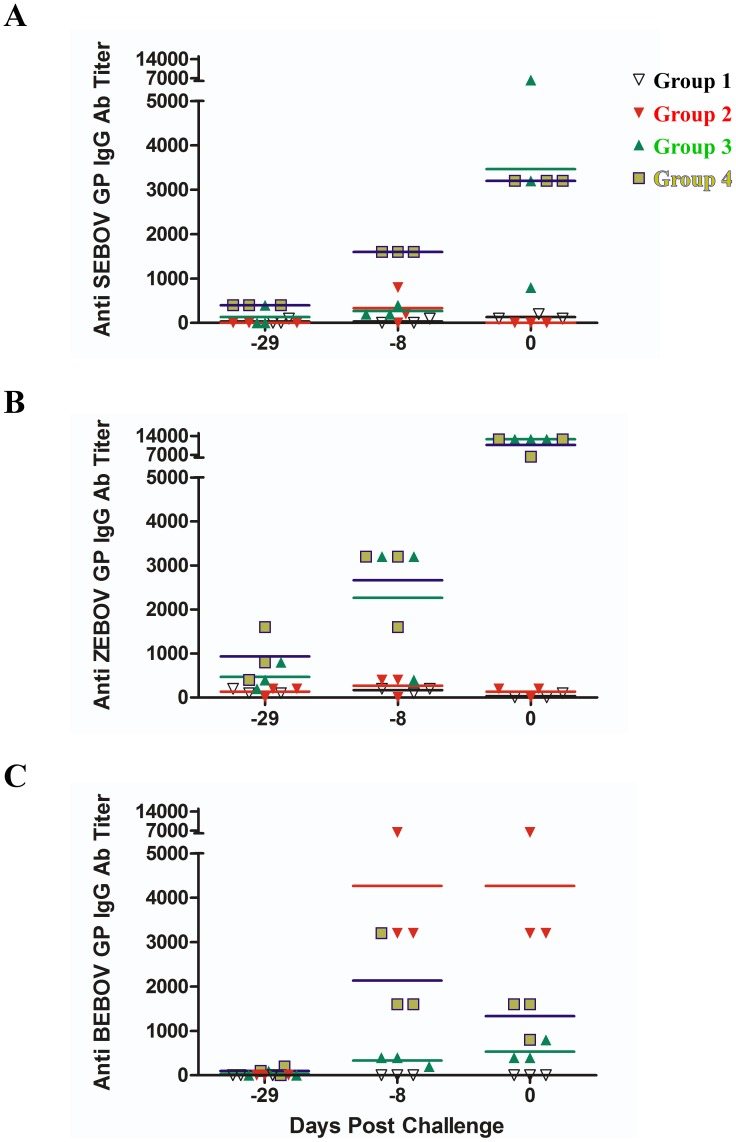
IgG antibody response to rVSV-filovirus-GP vaccination. Reciprocal endpoint dilution titers for IgG against SEBOV GP (A), ZEBOV GP (B), and BEBOV GP (C) were determined from serum samples in each Group at 29, 8, and 0 days before challenge. Group 1 (PBS only control, black), Group 2 (rVSV-BEBOV-GP only, red), Group 3 (rVSV-SEBOV-GP plus rVSV-ZEBOV-GP, green), and Group 4 (rVSV-SEBOV-GP only, then rVSV-ZEBOV-GP 14 days post, yellow/blue). Red **, p<0.01 (Group 2 vs Group 4), Red ***, p<0.001 (Group 2 vs Group 4), Blue **, p<0.01 (Group 3 vs. Group 4), and Blue >, p>0.05 (Group 3 vs Group 4).

To determine the humoral immune response to the different vaccination strategies (Fig. 1B and C), we tested the pre-challenge serum of the animals for immunoglobulin G (IgG) antibody titers that were cross-reactive for SEBOV GP (Fig. 2A), ZEBOV GP (Fig. 2B), or BEBOV GP (Fig. 2C) by enzyme-linked immunosorbent assay (ELISA). Mean reciprocal titers of IgG antibodies were calculated and are shown in Figure 2. As expected, we observed no antibody titers for Group 1 when tested against all three EBOV GPs (Fig. 2A, B, and C) whereas we detected only BEBOV GP cross-reactive IgG for Group 2 at day −8 pre-challenge and on the day of challenge (Fig. 2C, Day −8 and 0). Groups 3 and 4 were vaccinated with the same vaccine vectors but had different vaccination regimens (Fig. 1B and C). While the day 0 IgG titers for SEBOV GP (Fig. 2A, Day −8 and 0) and ZEBOV GP (Fig. 2B, Day −8 and 0) were similar between the groups there was a higher cross-reactive IgG titer for BEBOV GP in the cohort from Group 4 (Fig. 2C, Day −8 and 0). In addition, although BEBOV GP cross-reactive IgG titers were not as high as those elicited in Group 2, the vaccination regimen for Group 4 did elicit IgG antibodies which could recognize BEBOV GP.

### BEBOV challenge post-vaccination

**Figure 3:**
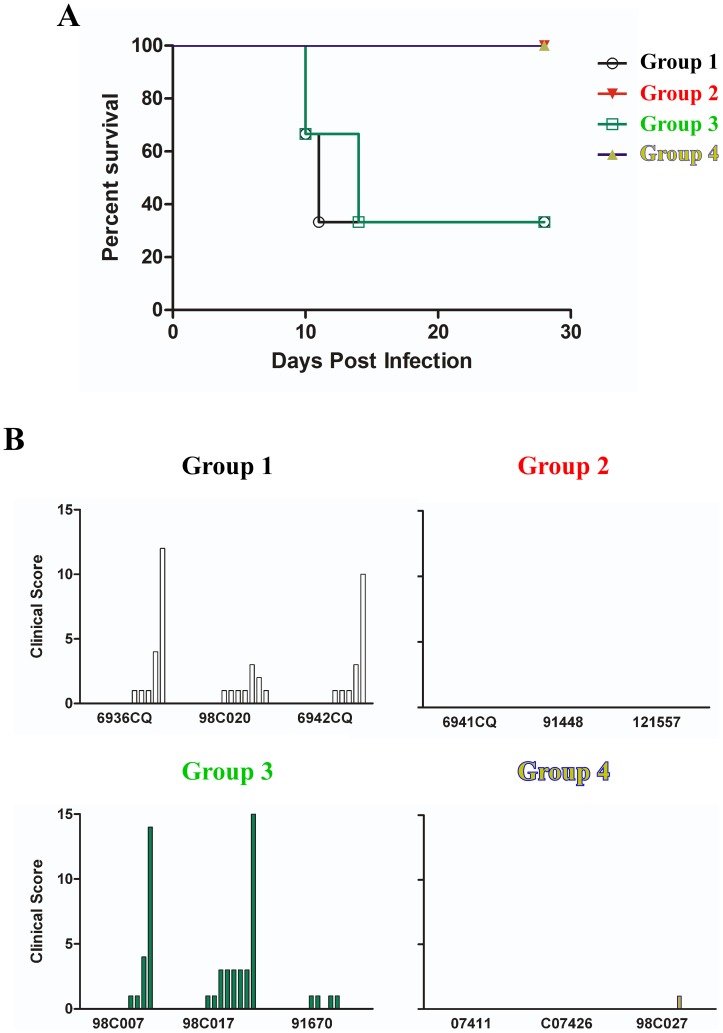
Group outcomes of BEBOV challenge. (A) Kapplan-Meier survival curve for each Group post BEBOV challenge. (B) Clinical scores for each individual within each Group after BEBOV challenge. Group 1 (PBS only control, black), Group 2 (rVSV-BEBOV-GP only, red), Group 3 (rVSV-SEBOV-GP plus rVSV-ZEBOV-GP, green), and Group 4 (rVSV-SEBOV-GP only, then rVSV-ZEBOV-GP 14 days post, yellow/blue). The x-axis represents clinical scores from Day 0 to Day 14 post challenge for each individual animal to show disease progression.

To date, studies have shown that the mortality rate for the cynomolgus macaque model after BEBOV challenge is between 66% and 75% [23], [25], whereas the SEBOV and ZEBOV models are 100% lethal [16]. To test whether we could induce cross-protection against BEBOV challenge after vaccinating with heterologous rVSV vaccines expressing SEBOV and ZEBOV GPs, we challenged all four groups of NHPs with a 1,000 pfu dose of BEBOV. The animals were closely monitored over the course of 28 days post-challenge for clinical signs of illness. Groups 2 and 4 were 100% protected against BEBOV (Fig. 3A), while Groups 1 and 3 each had two of the three animals succumb to BEBOV infection (Fig. 3A). For Group 1, animal 6936CQ succumbed on day 11 and animal 6942CQ succumbed on day 10 post-challenge (Table 1). In Group 3, animal 98C007 succumbed on day 10 and animal 98C017 from this group expired on day 14 post-challenge. Clinical scores were recorded each day post-challenge for each animal using a scoring system based on dyspnea, depression, recumbency, and rash. The clinical scores for each animal associated with the survival data as seen with animal 98C020 from Group 1 and animal 91670 from Group 3 each scoring lower than the non-surviving animals in their cohort (Fig. 3B).

**Table 1:** Clinical findings and viremia for NHPs challenged with BEBOV.

Animal	Group	Vaccine^a^	Signs Observed Between Day 0 and 28 after BEBOV challenge^b^	Serum Viremia^d^	Final Outcome
**6936CQ**	**1**	PBS	Fever (6, 10), Anorexia (7–11), Depression (7–11), Rectorrhagia (11), Lymphopenia (6, 10), Thrombocytopenia (10, 11), ALP→→ (10), ALP→→→ (14), AST→→ (10, 11), BUN→ (10), BUN→→ (11), GGT→ (11)	0/+(3), **5.6**/++(6), **6.5**/++(10)	Died on day 11
**98C020**	**1**	PBS	Anorexia (8–14), Depression (7–13), Lymphopenia (6), Thrombocytopenia (6, 10), ALP→ (10), AST→→ (10), AST→ (14), BUN→ (10), GGT→ (10)	0/+(3), **4.9**/++(6), **3.4**/++(10), 0/+(14)	Survived
**6942CQ**	**1**	PBS	Fever (10), Anorexia (7–10), Depression (7–10), Epistaxis (10), Lymphopenia (6), Thrombocytopenia (10), ALP→ (10), AST→→→ (10), BUN→ (10), GGT→ (10)	0/+(3), **4.2**/++(6), **6.5**/++(10)	Died on day10
**6941CQ**	**2**	rVSV-BEBOV-GP	Ø^c^	0/−	Survived
**91448**	**2**	rVSV-BEBOV-GP	Ø	0/−	Survived
**121557**	**2**	rVSV-BEBOV-GP	Ø	0/−	Survived
**98C007** *	**3**	rVSV Blend	Fever (6), Moderate rash (9–10), Anorexia (7–10), Depression (7–10), Lymphopenia (6), Thrombocytopenia (6)	**3.4**/++(6)	Died on day 10
**98C017**	**3**	rVSV Blend	Fever (6), Anorexia (7–14), Depression (7–14), Epistaxis (14), Lymphopenia (10), Thrombocytopenia (6, 10, 14), ALP→→ (6), ALP→→→(10, 14), BUN→ (10), BUN→→→ (14), GGT→ (10, 14)	**6.6**/+++(6), **5.1**/++(10), **6.1**/++(14)	Died on day 14
**91670**	**3**	rVSV Blend	Fever (6), Anorexia (7–10), Depression (7–10), Lymphopenia (6),	**1.7**/++(6), **2.6**/+(10)	Survived
**07411**	**4**	rVSV Prime-boost	Mild anorexia (9–11), ALP→ (10, 14), AST→→ (10), BUN→ (10)	0/+(6) 0/+(10)	Survived
**C07426**	**4**	rVSV Prime-boost	Ø	0/−	Survived
**98C027**	**4**	rVSV Prime-boost	Mild fever (6), Mild anorexia (9–10), ALP→ (10)	0/+(6)	Survived

*98C007 expired before sampling at day 10 could be achieved.

^a^ rVSV blend; rVSV-SEBOV-GP plus rVSV-ZEBOV-GP, rVSV Prime-boost; rVSV-SEBOV-GP first then rVSV-ZEBOV-GP 14 days after.

^b^ Days after BEBOV challenge are in parentheses. Fever is defined as a temperature more than 2.5°F over baseline or at least 1.5°F over baseline and ≥103.5°F. Moderate rash refers to petechiae coverage of more than 20% of the skin. Lymphopenia and thrombocytopenia are defined by a ≥35% drop in numbers of lymphocytes and platelets, respectively. (ALP) alkaline phosphatase, (AST) aspartate aminotransferase, (BUN) blood urea nitrogen, (GGT) gamma glutamyltransferase: 2- to 3-fold increase,→; 4- to 5-fold increase, →→; >5 fold increase, →→→.

^c^ No symptoms observed.

^d^ Days after BEBOV challenge are in parentheses. Viral load for each day is depicted as: log_10_ PFU/ml**/**qRT-PCR positive (+) or negative (−). +, ≤5 log_10_; ++, ≥6 log_10_; +++, ≥7 log_10_.

The signs of disease in response to BEBOV infection were more dramatic for the animals in Groups 1 and 3 when compared to the animals in the other two groups (Table 1). This observation correlates well with the fact that infectious virus was only isolated from the serum of all the animals in these groups after challenge (Table 1). Though reduced when compared to Groups 1 and 3, Group 4 had one animal (Table 1, 98C027) with very mild signs of disease and two of the animals (Table 1, 07411 and 98C027) were positive for viral genomes in serum as detected by qRT-PCR, whereas Group 2 had no signs of disease nor were the serum samples positive for viral genomes by qRT-PCR (Table 1).

### Neutralizing BEBOV GP antibody titers pre- and post-challenge with BEBOV

To further address the humoral response to rVSV-filovirus-GP vaccination and BEBOV challenge, we assessed sera for neutralizing activity against BEBOV GP. Neutralizing antibody titers were not detected in any animal before vaccination (Table 2, Pre-vaccination). None of the 3 animals in Group 4 showed any evidence of neutralizing antibodies after the prime vaccination (Table 2, Day −22). By the day of BEBOV challenge there were five animals that had modest neutralizing antibody titers (PRNT_50_ of 1∶40 to 1∶80) including all three animals from Group 2 and two of three animals from Group 4 (Table 2). Neutralizing antibody titers were also assessed for all animals at the study endpoint (day of death for animals that expired or day 28 for surviving animals). All animals that survived BEBOV challenge had PRNT_50_ titers ranging from 1∶40 to 1∶160 against BEBOV GP while with the exception of one control animal all macaques that succumbed had PRNT_50_ values below 1∶40 (Table 2).

**Table 2:** Reciprocal BEBOV GP serum neutralizing antibody titers at which 50% of rVSV-BEBOV-GP was neutralized.

Animal	Group	Vaccine^a^	Pre-Vaccination	Day −22^b^	Day 0^b^	Terminal^c^
**6936CQ** *	**1**	PBS	≤20	n.d.	≤20	≤20
**98C020**	**1**	PBS	≤20	n.d.	≤20	**40**
**6942CQ** *	**1**	PBS	≤20	n.d.	≤20	≤20
**6941CQ**	**2**	rVSV-BEBOV-GP	≤20	n.d.	**80**	**40**
**91448**	**2**	rVSV-BEBOV-GP	≤20	n.d.	**80**	**80**
**121557**	**2**	rVSV-BEBOV-GP	≤20	n.d.	**40**	**40**
**98C007** *	**3**	rVSV Blend	≤20	n.d.	≤20	≤20
**98C017** *	**3**	rVSV Blend	≤20	n.d.	≤20	**80**
**91670**	**3**	rVSV Blend	≤20	n.d.	≤20	**40**
**07411**	**4**	rVSV Prime-boost	≤20	≤20	**40**	**160**
**C07426**	**4**	rVSV Prime-boost	≤20	≤20	≤20	**80**
**98C027**	**4**	rVSV Prime-boost	≤20	≤20	**80**	**40**

*Succumbed to BEBOV challenge.

^a^ rVSV blend; rVSV-SEBOV-GP plus rVSV-ZEBOV-GP, rVSV Prime-boost; rVSV-SEBOV-GP first then rVSV-ZEBOV-GP 14 days after.

^b^ Days after BEBOV challenge, Day −22; day of boost.

^c^ See Table 1 for Terminal sample day of animals with a *; all others are from Day 28.

## Discussion

The emergence of BEBOV in 2007/08 and the recent outbreak in the summer of 2012 [3], [6] are events which underscore the lack of effective vaccines for combating new species of EBOV during outbreaks. While there are well characterized vaccines against ZEBOV and SEBOV [9], [10], [11], [12], [13], [14], [15], [16], [17], [18], [19], [20], [21], [22], [23], [24], [25], [26], [27], [28], [29], until this report, there were no vaccines directed specifically against BEBOV. Here, we have shown that the rVSV-BEBOV-GP vaccine can perform just as well against homologous challenge with BEBOV as seen in previous studies with the rVSV vaccines against homologous challenge with ZEBOV or SEBOV [12], [33]. In this study the animals in Group 2 lacked clinical signs of disease, were below the limits of detection for viremia by plaque assay and qRT-PCR (Table 1), and each animal in the cohort had a score of “0” on the clinical score scale (Fig. 3B) for the duration of the study; all results were similar to vaccination against homologous challenge with ZEBOV or SEBOV after vaccination with the appropriate rVSV-filovirus-GP vector.

As with previous studies [23], [25], we were interested in whether or not we could protect against a newly emerging EBOV species with vaccine vectors that were available at the time of an outbreak. Therefore, we used the BEBOV model and the rVSV-SEBOV-GP and rVSV-ZEBOV-GP vaccines available during the initial BEBOV outbreak. In an emergency intervention scenario, a vaccination schedule requiring a number of boosts over a long period of time is not practical. A rapid, single vaccination strategy is desired in this scenario. However, available data suggest that heterologous rVSV-filovirus-GP vaccines cannot always protect against challenge with a different species of EBOV [12], [25], [34] nor can a single, blended vaccination using multiple rVSV-filovirus-GP protect as seen in Group 3 in the current study (Fig. 3A, Table 1). The ability to cross-protect against BEBOV challenge using DNA/rAd-based SEBOV and ZEBOV GP vaccines [23], [25] encouraged us to use the rVSV-SEBOV-GP and rVSV-ZEBOV-GP as a vaccine strategy for cross-protection against BEBOV challenge. Data from a blended vaccine study using rVSV-SEBOV-GP, rVSV-ZEBOV-GP, and rVSV-MARV-GP suggest that there is potential for vector interference/competition between the EBOV vaccines in particular in regard to the effectiveness of the SEBOV vaccine in the blend [16]. Based on these data, we tested the blended approach (Group 3) but also employed a prime-boost strategy (Group 4) which allowed the rVSV-SEBOV-GP vaccine to induce an immune response to SEBOV-GP 14 days before vaccination with rVSV-ZEBOV-GP and subsequent BEBOV challenge 21 days after vaccination with the ZEBOV vaccine. We hypothesized that this prime-boost strategy with the rVSV platform would provide similar cross-protection when compared with the DNA and rAd-based approach [23], but with fewer doses (2 versus 5) and a much shorter vaccine regimen (36 days versus 518 days). Indeed, we were able to induce 100% protection against heterologous BEBOV challenge with the Group 4 vaccination regimen (Fig. 3A, Table 1).

While 100% and 75% protection was achieved between our study and the previous BEBOV cross-protection studies [23], [25], the immunity without any detectable viremia generally seen against homologous challenge [12], [15], as noted in Group 2, was not achieved by the Group 4 vaccine regimen. This observation was also seen in the previous BEBOV studies [23], [25] with detectable viremia by qRT-PCR and very mild clinical signs of disease reported (Table 1). The difference between Group 4 and Groups 1 and 3 is clear with animals in each of the latter groups succumbing to infection (Fig. 3A, Table 1), showing more severe clinical signs of disease, higher levels of viral RNA, and detectable circulating infectious virus (Table 1).

It is interesting when comparing the differences between Groups 3 and 4 (where the only deviation was in the regimen used) that animals in Group 4 were protected from severe disease while the animals in Group 3 experienced similar signs of severe disease as the control animals in Group 1 (Table 1). While the circulating level of BEBOV cross-reactive GP IgG from Group 4 was not as high as from the homologous vaccine animals (Group 2) (Fig. 2C, p<0.01 at Day −8) the circulating cross-reactive BEBOV GP IgG from Group 4 was higher than the level from Group 3 (Fig. 2C, p<0.01 at Day −8). In contrast to the blended strategy, the prime-boost regimen was able to generate a greater cross-protective immunity which was associated with the higher cross-reactive BEBOV GP IgG. This is different from the DNA/rAd vaccine strategy where there were no cross-reactive BEBOV GP IgGs detected, although there was a cellular immune response by CD4^+^ and CD8^+^ T-cells [23]. This observation may not be too surprising as the DNA/rAd vaccines have been shown to elicit robust cellular immunity [23], [35] while it has recently been demonstrated that antibodies correlate with protection against ZEBOV infection using the rVSV-based vaccine platform [28]. In fact, single immunization with either rVSV-ZEBOV-GP or rVSV-CIEBOV was able to generate some cross-reactive BEBOV-GP IgG, although higher for the CIEBOV vaccine group. However, these antibody responses did not correlate with 100% protection [25].

While it was reported that antibodies are necessary for protection using the rVSV-ZEBOV-GP vaccine [28], the neutralizing antibody titers are not very robust when compared to responses induced by vaccines against other highly pathogenic viruses such as Nipah virus [36]. However, this previous work shows that even low to modest levels of neutralizing antibodies appear to be important for protection of NHPs against ZEBOV as a productive immune response was evidenced by increased titers after virus challenge [28]. While ZEBOV is uniformly lethal in cynomolgus macaques, the mortality rate for BEBOV in cynomolgus monkeys is 66 to 75% with a prolonged time to death compared to ZEBOV [23], [25]. This difference in disease pathogenesis confounds a definitive conclusion in the current study when using the development of neutralizing antibodies to determine a productive immune response to the different vaccine regimens between Groups 3 and 4. This is reflected in Table 2 where we observed an increase in neutralizing antibody titer post-challenge for the non-vaccinated Group 1 survivor (98C020), the rVSV blend Group 3 survivor (91670), and for the animal that succumbed in Group 3 (98C017). The Group 3 animal (987C017) succumbed on Day 14 which may account for the presence of antibodies in this case as the disease course was further delayed allowing for an antibody response, although not sufficient enough for protection. On the surface, the results in Table 2 appear to be the same for animal 98C017 from Group 3 and animal C07426 from Group 4 when looking at the antibody titers, although one animal succumbed and the other animal survived BEBOV challenge. While comparing these two animals is difficult, we believe the Day 0 titers shed light on the differences between responses to the vaccine regimens as there were no neutralizing BEBOV GP antibody titers for any animal in Group 3 at the day of BEBOV challenge while two of three Group 3 animals had neutralizing BEBOV GP antibody titers at the day of challenge (Table 2, Day 0). All antibody data taken together, it is clear that a prime vaccination with rVSV-SEBOV-GP and subsequent boost vaccination with rVSV-ZEBOV-GP produces higher levels of cross-reactive BEBOV GP IgG than the blended vaccination approach (Fig. 2C, Group 3 versus 4) and a pre-challenge neutralizing BEBOV GP antibody titer (Table 2, Day 0). It is also evident that these anti-BEBOV binding and neutralizing antibodies are associated with protection in a prime-boost regimen versus a single immunization with a single heterologous rVSV-EBOV-GP vector [25].

Comparison between our study and the previous two BEBOV cross-protection studies are similar on the surface as each has shown some measure of cross-protection with animals displaying some mild signs of illness and low level viremia by qRT-PCR. However, there are differences in the challenge doses used in each study. Specifically, our study used a 1,000 PFU challenge dose, the DNA/rAd study used 1,000 TCID_50_ [23], and the rVSV-filovirus-GP single immunization study used 10,000 TCID_50_ [25]. Here, we measured levels of infectious virus and viral RNA where only viral RNA was assessed to determine viremia in the previous studies [23], [25]. This makes it difficult to compare infectious viremia among the studies. However, we can compare the viremia reported by detection of BEBOV genomes among the studies. In the previous studies, the viremia detected in macaques that succumbed to BEBOV infection by qRT-PCR did not reach 6 log_10_ genome equivalents [23], [25]_,_ while all animals in Group 1 and Group 3 of the current study exceeded 6 log_10_ during the course of disease (Table 1). Based on these data it appears that our study may have had a higher infectious challenge dose and yet we were still able to provide cross-protection against BEBOV challenge with the prime-boost strategy using vaccines that were available at the time of the original BEBOV outbreak [3].

In this study we have shown that a rVSV-BEBOV-GP vector can protect against homologous challenge with the newest species of EBOV, BEBOV, and that a prime-boost strategy with the rVSV-SEBOV-GP and rVSV-ZEBOV-GP vectors, available at the time BEBOV emerged, is capable of providing cross-protection against BEBOV challenge. We propose that this condensed, prime-boost vaccine regimen of available heterologous rVSV-filovirus-GP vaccines should be considered as a paradigm for controlling newly emerging EBOV species. EBOV has recently re-emerged with a potential new species as filovirus-like RNA was isolated from dead bats in Spain [37]. In addition, an outbreak of REBOV recently occurred in pigs in the Philippines [38], [39], [40] and further studies have shown that ZEBOV can infect pigs [41], [42]. In light of these observations and the increasing number of filovirus outbreaks over the past decade, it would be prudent to have a strategy in place which could be used to immediately respond to an outbreak of a new EBOV species while a new homologous rVSV vaccine vector was being developed and produced. The typical period of time between vaccination and challenge for these vaccines in NHPs is 28 days although recently it has been reduced to 21 days (TWG, unpublished data). From this study we could potentially have the population surrounding an epicenter of a newly emerged filovirus protected within 35 days. In addition, we cannot rule out some post-exposure utility for the population, as these vectors have shown post-exposure potential [33], [43]. While we used single antigenic rVSV-filovirus vaccines in this study, there are rVSV-filovirus vectors that can express multiple filovirus antigens from different EBOV species which have shown improved cross-protective efficacy in guinea pigs [44]. Ideally, a single vaccination capable of immunizing against all EBOV species known to cause human disease would be preferred for a quick response to an outbreak of EBOV; however, at the moment it appears that a prime-boost strategy may be the best approach for broad coverage. Perhaps an approach using the prime-boost strategy used here with single antigenic vectors plus the multiple antigenic vectors would enhance cross-protection to a point where immunity with a lack of mild clinical signs and detectable low level viremia could be achieved.
